# The Effects of Dandelion Polysaccharides on Iron Metabolism by Regulating Hepcidin *via* JAK/STAT Signaling Pathway

**DOI:** 10.1155/2021/7184760

**Published:** 2021-01-02

**Authors:** Feng Ren, Yingying Yang, Kaixuan Wu, Tiesuo Zhao, Yinghao Shi, Moxuan Song, Jian Li

**Affiliations:** ^1^School of Basic Medical Sciences, Xinxiang Medical University, Xinxiang 453003, China; ^2^School of Nursing, Xinxiang Medical University, Xinxiang 453003, China; ^3^School of Forensic Medicine, Xinxiang Medical University, Xinxiang 453003, China

## Abstract

Recent studies have claimed that iron overload was correlated with the risk of hepatocellular carcinoma (HCC), and our previous studies have also demonstrated that dandelion polysaccharide (DP) suppressed HCC cell line proliferation via causing cell cycle arrest and inhibiting the PI3K/AKT/mTOR pathway, but the effect of DP on metabolism is still not very clear. Here, we aim to clarify the effects of DP on iron metabolism and the underlying mechanism. In this study, we found that DP could reduce iron burden in hepatoma cells and grafted tumors. Hepcidin is a central regulator in iron metabolism. We confirmed that the expression of hepcidin in HCC tumor tissues was significantly higher than that in the adjacent nontumor tissues. The expression of hepcidin was downregulated in the liver of mouse model treatment with DP, as well as in hepatoma cells. Moreover, RNA sequencing and western blot data revealed that DP inhibited the IL-6-activated JAK-STAT signaling pathway. In summary, our results revealed that DP might be a new potential drug candidate for the regulation of iron burden and the treatment of HCC.

## 1. Introduction

Primary liver cancer is the sixth most common cancer and the third most common cause of cancer-related deaths around the world. Hepatocellular carcinoma (HCC) accounts for 85%-90% of all primary liver cancers, and its overall 5-year survival rate is less than 16% [1, 2]. Iron is an essential component for life, and the homeostasis of iron is precisely governed in mammals. However, accumulating evidence suggest a direct correlation between excessed iron burden and increased risk of developing cancer [3–5], especially for HCC [6–8]. Iron chelators are considered as an adjuvant drug in tumor therapy [9–11].

Hepcidin, a central regulator in iron metabolism, is prominently synthesized and secreted in the liver. Ferroportin, the only known exporter of intracellular iron, is the receptor of hepcidin. Hepcidin binds and induces degradation of ferroportin and inhibits iron absorption from the duodenum, iron release from macrophages, and stored iron egress from hepatocytes [12, 13]. High expression of hepcidin was found in tumor tissues rather than in adjacent nontumor tissues, which was confirmed to be associated with carcinogenesis and tumor development [4, 14–17]. The JAK/STAT signaling pathway plays crucial roles in many cell progresses, including cell proliferation and immune response. Many studies have reported that JAK/STAT signaling is aberrantly activated in HCC and activated JAK/STAT pathway causes dysregulation of its many downstream target genes correlated with proliferation, immune, invasion, and metastasis [18, 19]. In addition, hepcidin activates the JAK/STAT pathway through Jak2 activation and transcription factor Stat3 phosphorylation [20].

Dandelion is a traditional Chinese medicinal herb belonging to the Asteraceae family and is widely used as a diuretic, anti-inflammatory, and antioxidant compound [21, 22]. However, the anticancer effects and the underlying mechanisms of dandelion polysaccharides (DP) on HCC via affecting iron metabolism remain unknown.

A growing body of evidence has suggested that dandelion possessed anticancer effects against breast cancers, gastric cancers, and prostate cancers [23–25]. In our previous research, we showed that dandelion polysaccharide (DP) markedly inhibited the proliferation and growth of HCC cancer cells [26]. Presently, we discovered that DP decreased iron burden in tumor tissues of Hepa1-6 and H22 tumor-bearing mice and hepatoma cancer cells. Moreover, DP could regulate hepcidin expression *in vitro and in vivo* via inhibiting the phosphorylation of JAK2 and STAT3. Taken together, our data demonstrated that DP suppressed HCC growth through regulating the expression of hepcidin by inhibiting the activity of the JAK/STAT signaling pathway.

## 2. Materials and Methods

### 2.1. Cell Lines and Cell Culture

Human hepatocellular carcinoma HepG2 and Huh7 cell lines were purchased from Stem Cell Bank of Chinese Academy of Sciences. Mouse hepatocellular carcinoma Hepa1-6 and H22 cells were obtained from China Center for Type Culture Collection. All cells mentioned above were maintained at 37°C and incubated in 5% CO_2_. All cells were cultured in DMEM (Hyclone, Logan, USA) supplemented with 10% fetal bovine serum (Hyclone, Logan, USA). DP with purity of >98% was obtained from Ci Yuan Biotechnology Co., Ltd. Shanxi (Xian, China). For our experiments, HepG2 and Huh7 cells were treated at an increasing concentration of DP (0, 100, 200, or 400 mg/L) on different time points (0, 24, 48, 72, or 96 h). The optimal induction concentration and time of DP for decreasing hepcidin expression were determined.

### 2.2. Animals and Treatment

Balb/C mice were obtained from Vital River Laboratory Animal Technology Co., Ltd. (Beijing, China). The mice were subcutaneously injected with Hepa1-6 cells or H22 cells (2 × 10^6^ cells per mouse) and randomly separated into two groups: the control group and the DP group. The DP group was i.p. injected with 200 mg/kg of DP once daily for 14 days [26]. The control group was treated with 0.9% saline via intraperitoneal injection. At last, the liver and tumor of mice were harvested for further analysis. The procedures were approved by the Animal Care Committee of Xinxiang Medical University.

### 2.3. RT-PCR Analysis

The total RNA of cells was isolated with TRIzol (Invitrogen, Carlsbad, USA). cDNA was synthesized using Prime-Script™ RT Master Mix according to the supplier's protocol. The mRNA expression was quantified by using RT-PCR analysis with SYBR Green qPCR master mix (Qiagen, Hilden, Germany). The primers for quantifying hepcidin were 5′-cctgaccagtggctctgttt-3′ and 5′-cacatcccacactttgatcg-3′. The primers for quantifying GAPDH were 5′-aatcccatcaccatcttcca-3′ and 5′-cctgcttcaccaccttcttg-3′.

### 2.4. RNA Sequencing and Bioinformatics Analysis

We conducted RNA sequencing for HepG2 cells treated with DP (200 mg/L, 48 h) and their control samples on BGISEQ-500 sequencing platform. Bowtie 2 and RSEM were separately used to map the clean reads to reference the transcripts and calculate the gene expression level for each sample. The RNA-seq expression levels were normalized using the Fragments Per Kilobase of transcript per Million mapped reads. We used DEGseq algorithms to detect the differentially expressed genes (DEGs), and the genes that were differentially expressed in HepG2 cells treated with DP and their control samples with *q* value < 0.001 were selected as DEGs. Pathway enrichment analysis for DEGs was conducted using a web server, KOBAS V3.0.

### 2.5. Western Blotting

Cells were lysed with RIPA lysis buffer. Tumor tissue samples were pelleted on ice. Protein lysates were subjected to western blot according to the method described previously [26]. All primary antibodies in the present study were listed as follows: hepcidin (Abcam, Cambridge, UK); STAT3, p-STAT3, JAK2, and p-JAK2 (CST, Beverly, USA); and *β*-actin (Santa Cruz, USA).

### 2.6. Measurement of Intracellular Iron

The intracellular labile iron pool (LIP) was determined using a fluorescence technique with Fe sensor calcein. The HepG2 and Huh7 cells were cultured on coverslips and treated with DP at a concentration of 200 mg/L for 48 hours. Then, the cells were washed and incubated with 50 *μ*M calcein-AM (Sigma-Aldrich) for 30 min at 37°C. The excess calcein-AM was washed off with PBS. The immunofluorescent signals were detected by using a fluorescence microscope (*λ*_exc_ = 490 nm, *λ*_em_ = 515 nm) (Leica Microsystems, Wetzlar, Germany). The calcein-AM fluorescence was quenched by intracellular iron.

### 2.7. Immunofluorescence (IF)

HepG2 and Huh7 cells were fixed with 4% paraformaldehyde at 4°C for 10 min and incubated in 0.3% Triton for 30 min. After blocking with 5% goat serum for 30 min at room temperature, the cells were incubated with anti-hepcidin (Abcam, Cambridge, UK) overnight at 4°C, and then, cells were incubated with secondary antibody (Proteintech, USA) for 2 h. DAPI was used to stain the nucleus. The immunofluorescent signals were detected by a fluorescence microscope (Leica Microsystems, Wetzlar, Germany).

### 2.8. Prussian Blue Staining

Prussian blue staining was used to detect iron depositions in tumors. The tumor tissues were incubated in Perls' reagent with 5% potassium ferrocyanide and 5% hydrochloric acid for 15 min at room temperature. Images of the tissues were captured using a Nikon microscope.

### 2.9. Immunohistochemistry

Approximately 3 *μ*m thick liver sections were incubated with anti-hepcidin (Abcam, Cambridge, UK) overnight at 4°C. After washing with PBS, the sections were incubated with the secondary antibodies at room temperature for 2 hours.

### 2.10. Statistical Analysis

The data were shown as the mean ± SD. The groups were compared by one-way analysis of variance, and LSD *t*-test was utilized for multiple comparisons using SPSS 21.0 (IBM SPSS for Windows, Version 21.0; IBM Corporation, Armonk, NY, USA). *P* < 0.05 was considered significant.

## 3. Results

### 3.1. DP Regulates Iron Burden in Hepatoma Cells and Grafted Tumors

**Figure 1:**
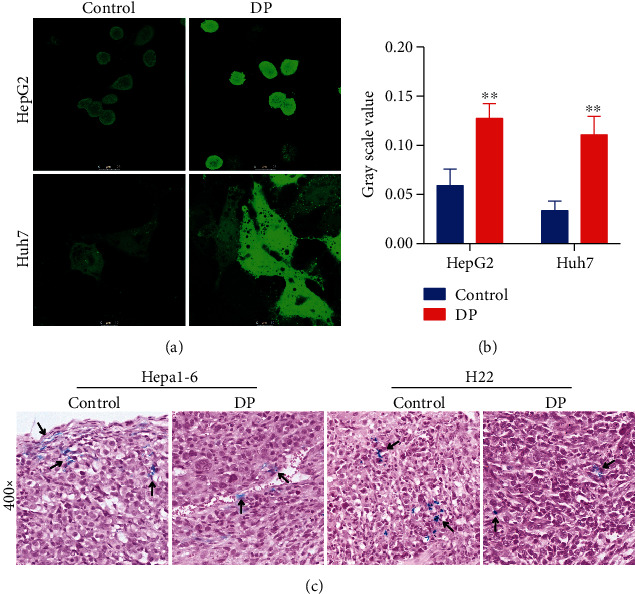
DP regulates iron content in hepatoma cells and grafted tumors. (a, b) Fluorescence staining of metal-sensitive probe calcein showed labile iron in HepG2 and Huh7 cells. DP decreased the labile iron content in both cells. (c) Perls' Prussian blue staining was performed to observe iron deposits in tumor tissues.

To evaluate the effect of DP in regulating iron content, the intracellular LIP of hepatoma cells was determined using calcein-AM assay. The HepG2 and Huh7 cells were selected and treated with 200 mg/L DP or vehicle for 48 hours. As shown in Figures 1(a) and 1(b), the DP significantly decreased labile iron in HepG2 and Huh7 cells compared with the DMSO control groups (*P* < 0.01).

Prussian blue staining was performed to detect the iron depositions in tumor tissues of mice. Mice bearing established Hepa1-6 or H22 tumors were randomly separated into 2 groups separately, including the DP group (treated with DP for 14 days) and vehicle (0.9% saline). As demonstrated in Figure 1(c), the accumulation of tissue iron (the arrow) was ameliorated in the tumor sections derived from the DP-treated mice compared with the control group.

### 3.2. Hepcidin Is Upregulated in HCC

**Figure 2:**
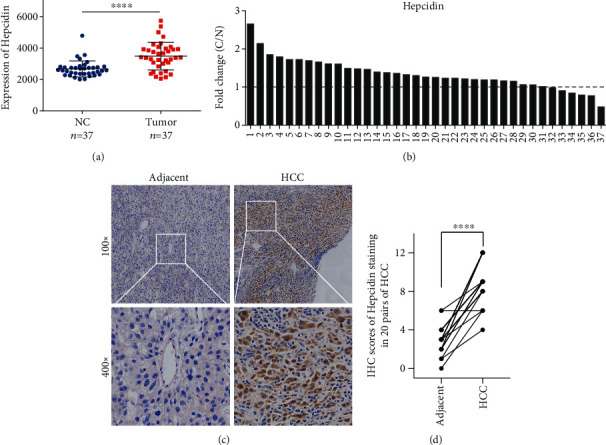
Hepcidin is upregulated in HCC. (a, b) Hepcidin expression was analyzed using the GEO dataset GSE57957. ^∗^Significant difference compared with the normal group (*P* < 0.05). (c, d) Representative IHC images of hepcidin staining in HCC tumor or adjacent nontumor tissues were presented.

We assayed the expression level of hepcidin in HCC using the GSE57957 dataset. The results confirmed that the expression of hepcidin in tumor tissues was higher than that in the adjacent nontumor tissues from 37 pairs of HCC samples (Figures 2(a) and 2(b)). Moreover, we discovered a higher positive rate of hepcidin in HCC tumor tissues on the basis of IHC staining (Figures 2(c) and 2(d)).

### 3.3. DP Decreases Hepcidin Level *In Vitro*

**Figure 3:**
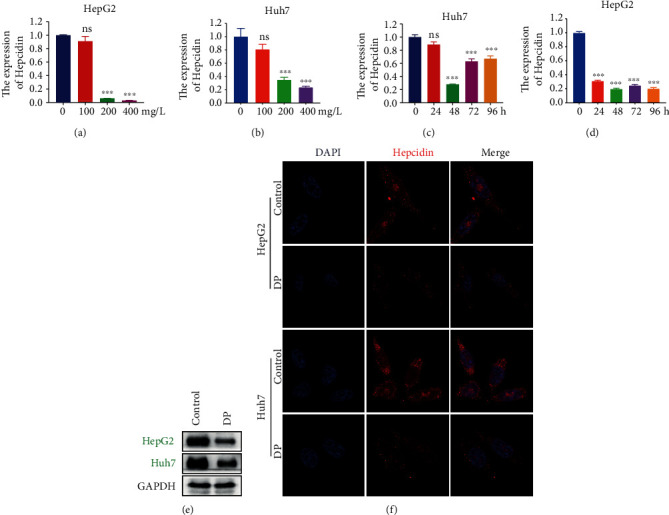
DP decreases the expression of hepcidin in hepatoma cells. (a, b) RT-PCR analysis showed the hepcidin mRNA level in HepG2 and Huh7 cells treated with various concentrations of DP for 48 h. (c, d) RT-PCR analysis showed the hepcidin mRNA level in HepG2 and Huh7 cells treated with 200 mg/L DP for various times. (e) Protein expression levels of hepcidin were analyzed by western blot in HepG2 and Huh7 cells cultured in 200 mg/L DP for 48 h. (f) IF assay was used to detect hepcidin in HepG2 and Huh7 cells treated or untreated with DP. Hepcidin was stained with red color, whereas the nuclei stained with DAPI were blue.

To investigate whether DP decreases the expression of hepcidin, the master regulator of iron homeostasis, we firstly evaluated the effects of DP on hepcidin gene expression in HepG2 and Huh7 cells. The RT-PCR result showed that DP significantly reduced the expression of hepcidin mRNA level in cells incubated with 200 and 400 mg/L DP for 48 h (Figures 3(a) and 3(b)). Next, we examined the time course of the effects of 200 mg/L DP on hepcidin expression. The results suggested that the decreased peak point of hepcidin mRNA in HepG2 and Huh7 cells was at 48 h (Figures 3(c) and 3(d)). The protein levels of hepcidin in HepG2 and Huh7 cells were tested upon the treatment of DP (200 mg/L) for 48 h. Similar to the RT-PCR result, western blot analysis and IF staining also verified that hepcidin protein level was markedly reduced by DP treatment (Figures 3(e) and 3(f)).

### 3.4. DP Decreases the Expression of Hepcidin *In Vivo*

**Figure 4:**
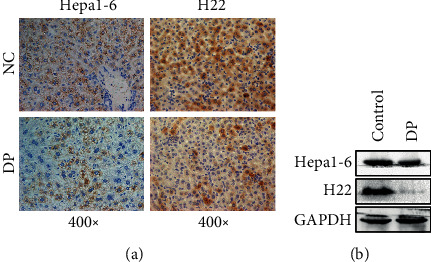
DP decreases the expression of hepcidin *in vivo*. (a) Immunohistochemical analysis of tumor tissues from mice bearing Hepa1-6 and H22 cancer cells. The tumor sections were subjected to IHC staining using an antibody against hepcidin. The magnification was ×400. (b) The expression level of hepcidin was analyzed using western blot in tumor tissues from Hepa1-6 and H22 tumor-bearing mice.

To further study the effect of DP on hepcidin expression *in vivo*, the Balb/C mice bearing established Hepa1-6 and H22 tumors were i.p. injected with DP at a dose of 200 mg/kg per day for 14 days. The protein level of hepcidin in mouse liver was measured by western blot and IHC. Our results indicated that the hepcidin protein level was significantly reduced compared with that in the control group (Figures 4(a) and 4(b)).

These results revealed that DP administration could effectively downregulate hepcidin level *in vitro* and *in vivo*.

### 3.5. DP Regulates Hepcidin Expression *via* the JAK/STAT Pathways *In Vitro*

**Figure 5:**
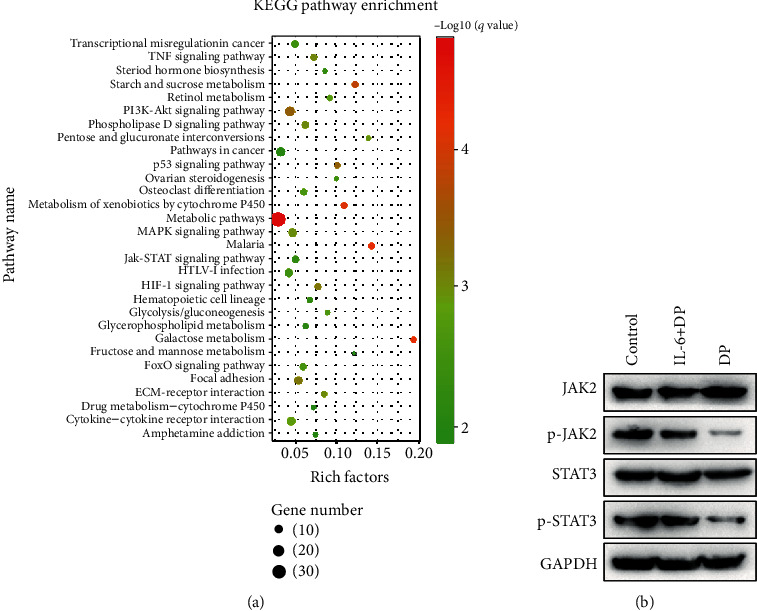
DP inhibits the activation of the JAK/STAT pathway. (a) Pathway enrichment analysis of DEGs on the basis of the KEGG database. The 30 most enriched pathways are displayed. (b) The expression of JAK2, p-JAK2, STAT3, and p-STAT3 proteins was analyzed using western blot.

To determine the effect of DP on hepcidin expression, RNA-seq was conducted to detect the expression of DP on the transcriptomic profile in HepG2 cells. Pathway analysis showed that DP treatment altered several signaling pathways, including PI3K/AKT, JAK-STAT, MAPK, and HIF-1 pathways (Figure 5(a)). As we know, the JAK/STAT signaling pathway is important for the transcription of hepcidin. Therefore, we analyzed the expression of key factors involved in the pathway in HepG2 cells after DP treatment. Our results showed that the expression of p-JAK2 and p-STAT3 was significantly decreased in the DP group compared with the control group. However, pretreatment with 20 ng/mL of interleukin 6 (IL-6) (a specific activator of STAT3) resulted in a significant increase in the expression of p-JAK2 and p-STAT3 proteins (Figure 5(b)).

Collectively, these results shown here indicated that DP inhibited the expression of hepcidin by targeting IL-6-induced JAK/STAT signaling pathways *in vitro*.

## 4. Discussion

The liver is the major organ for iron storage in the human body. Clinical studies have indicated that intrahepatic iron overload was positively correlated with the risk of developing HCC [7, 8]. Recently, some studies demonstrated that patients with thalassemia and hereditary hemochromatosis, which include liver iron overload, have a high risk for developing HCC [27–29]. HCC patients with portal iron overload have a considerably lower 5-year overall survival rate than those with negative portal iron score [30, 31]. Therefore, iron reduction therapy maybe a promising adjuvant therapy for treating HCC. Previously, our studies indicated that DP significantly inhibited the proliferation of HCC cells. In the present research, we verified that DP decreased labile iron in HepG2 and Huh7 cells and the iron depositions were considerably reduced in tumor tissues of mice bearing Hepa1-6 and H22 tumors compared with the control group after treatment with DP.

Systemic iron homeostasis is regulated by the hepcidin-ferroportin axis under normal physiological conditions, through suppressing iron absorption from the duodenum and iron egress from macrophages and hepatocytes [32, 33]. Ferroportin is the only known cellular iron exporter. The primary molecular action of hepcidin on regulating iron metabolism lies in hepcidin binding and inducing the degradation of ferroportin. As previously reported, our results confirmed that the level of hepcidin in tumor tissues was higher than that in adjacent nontumor tissues on the basis of GSE57957 dataset analysis and IHC staining of HCC tumor tissues. In the current study, we also demonstrated that following DP treatment, the level of hepcidin was downregulated *in vitro and in vivo*, which may be one of the reasons why DP diminished tumor iron burden and inhibited tumor growth.

Hepcidin transcription is regulated by BMP/SMAD and JAK/STAT pathways in response to inflammatory mediators and the erythropoietic pathway. The JAK/STAT signaling pathway is important in inflammation-induced hepcidin expression [34]. In this work, the RNA-seq data revealed that DP altered the JAK/STAT signaling pathway in HepG2 cells. Western blot results showed that DP administration markedly downregulated the levels of p-JAK2 and p-STAT3 in HepG2 cells. Given that IL-6 plays a key role in regulating inflammatory hepcidin expression by activating STAT3 [35, 36], we examined the roles of DP in the expression of JAK2, p-JAK2, STAT3, and p-STAT3 in HepG2 hepatoma cells treated with IL-6. The data indicated that pretreatment of HepG2 cells with IL-6 significantly recovered the protein levels of p-JAK2 and p-STAT3. Thus, the suppression of IL-6-induced JAK-STAT signaling pathway may be a possible mechanism involved in DP inhibiting hepcidin expression.

To conclude, we for the first time showed that DP regulated iron burden in hepatoma cells and grafted tumors by decreasing hepcidin expression *in vitro* and *in vivo*. The mechanism of DP in decreasing hepcidin expression relies on the suppression of IL-6-induced JAK/STAT signaling pathway. Therefore, our studies provide a novel insight into the anticancer effects of DP on HCC. Collectively, these findings highlighted DP as an innovative candidate for the treatment of HCC.

## Data Availability

The data used to support the findings of this study are included within the article.
